# Sunshine, Sea, and Season of Birth: MS Incidence in Wales

**DOI:** 10.1371/journal.pone.0155181

**Published:** 2016-05-16

**Authors:** Lloyd D. Balbuena, Rod M. Middleton, Katie Tuite-Dalton, Theodora Pouliou, Kate Elizabeth Williams, Gareth J. Noble

**Affiliations:** Swansea University Medical School, Swansea, Wales, United Kingdom; University of Queensland, AUSTRALIA

## Abstract

Maternal sun exposure in gestation and throughout the lifetime is necessary for vitamin D synthesis, and living near the sea is a population level index of seafood consumption. The aim of this study was to estimate the incidence rate of multiple sclerosis (MS) in Wales and examine its association with sun exposure, coastal living, and latitude. The study used a database of MS hospital visits and admissions in Wales between 2002 and 2013. For the 1,909 lower layer super output areas (LSOAs) in Wales, coastal status, population, longitude/latitude, and average sunshine hours per day were obtained. Age-specific and age-standardised MS incidence were calculated and modelled using Poisson regression. The distribution of births by month was compared between MS cases and the combined England and Wales population. There were 3,557 new MS cases between 2002 and 2013, with an average annual incidence of 8.14 (95% CI: 7.69–8.59) among males and 12.97 (95% CI: 12.44–13.50) among females per 100,000 population. The female-to-male ratio was 1.86:1. For both sexes combined, the average annual incidence rate was 9.10 (95% CI: 8.80–9.40). All figures are age-standardized to the 1976 European standard population. Compared to the combined England and Wales population, more people with MS were born in April, observed-to-expected ratio: 1.21 (95% CI: 1.08–1.36). MS incidence varied directly with latitude and inversely with sunshine hours. Proximity to the coast was associated with lower MS incidence only in easterly areas. This study shows that MS incidence rate in Wales is comparable to the rate in Scotland and is associated with environmental factors that probably represent levels of vitamin D.

## Introduction

Several non-genetic factors including Epstein-Barr virus, insufficient sun exposure, and smoking are reported to increase the risk for multiple sclerosis [1]. With the UK having one of the highest prevalence rates for MS in Europe, [2]it is important to refine and update the incidence rate of this degenerative disease. Scotland has a prevalence rate (255 per 10^5^)[2]that is higher than the rates both in England (199.9 per 10^5^) and Wales (168 per 10^5^)[3]. Relatively few studies reported age- and sex-specific incidence rates [3, 4]and we are unaware of a study covering the entire Welsh nation.

It is theorised that the variation in MS prevalence by geography is due to environmental factors acting on genetic predispositions [5]. Vitamin D obtained from sunlight and the diet is converted by the body into the metabolite 1,25-dyhydroxyvitamin D which activates the vitamin D receptor (VDR) in cells [6]. In the brain, 1,25-dihydroxyvitamin D has direct and indirect effects on T-cell lymphocytes, modulating the immune system’s inflammatory response [7].With sufficient vitamin D, the balance between T-helper lymphocytes type 1 and type 2 is stabilized. By contrast, vitamin D deficiency is associated with having more disease-causing T-cells at the expense of regulatory cells [8]. This process is shared with other autoimmune diseases such as rheumatoid arthritis, inflammatory bowel disease, ulcerative colitis, and lupus erythematosus [9, 10].

Although the effects of sun exposure, latitude and month of birth overlap, month of birth is an index of vitamin D availability in gestation [11]while latitude probably reflects life-long exposure to UV radiation [12]. Sun exposure is the primary source of vitamin D synthesis in humans and its relevance to MS is well-studied. The critical periods for sunshine exposure have been variously reported as infancy and early years [12]to the entire childhood and adolescence [13]. Immigration from a low to a high risk area before age 15 (but not after) was associated with higher MS risk [14].

According to a systematic review, 16 studies found an excess of MS births in April or spring. In England and Scotland, MS births were higher in April or May than in the rest of the year, compared to the general population [10]. With vitamin D deficiency as the hypothesized cause of higher MS birth rates in spring, it is surprising that no association of 25OHD levels and risk of multiple sclerosis was found [15]. Using a Mendelian randomization method, Mokry and colleagues [16]reported that alleles associated with decreased 25OHD increased the odds of MS, indicating that vitamin D insufficiency is probably a causal agent for MS. Overall, the preponderance of evidence, a dose-response relationship, and biological plausibility all support a causal interpretation [6].

The correlation of MS prevalence with latitude is well-replicated. Studies from the US, Japan, and Australia/New Zealand reported that greater distance from the equator correlated with higher numbers of cases [17, 18]. The most striking effect is seen in temperate Tasmania where MS prevalence is seven times higher than in tropical northern Queensland [19]. By contrast, the “latitude gradient” was not observed in Norway and in France [20] and a SW-NE gradient was reported in the latter [21]. Latitude probably reflects levels of UV and vitamin D. It could however be confounded by the genetic similarity of people living in the same place [3]. One way of teasing apart genes and environment is by accounting for the distribution of the HLA-DRB1 allelle (the main genetic component for MS) when mapping MS prevalence by latitude. Simpson and colleagues reported that the association of MS with latitude persists after controlling for HLA-DRB1[22].

The influence of dietary vitamin D insufficiency or supplementation on MS is less widely studied. Circumstantial evidence from Norway showed a lower incidence of MS in coastal fishing areas as compared with inland areas [23]. It is proposed that a diet rich in fatty seafood augments UV levels [20]in the winter. However, seafood and dietary sources are not deemed adequate to meet the optimal 25OHD serum level of 75 nmol/l level [24]. A review of clinical trials reported that high-dose vitamin D supplementation was not associated with a decreased relapse rate for MS patients [25].

Our objectives in this study were to estimate age and sex specific incidence of MS in Wales and to assess its relation with sunshine hours, latitude, living by the coast, and month of birth.

## Materials and Methods

### Data sources

The data for this project were collated from various sources. Data on MS episodes came from the Secure Anonymised Information Linkage (SAIL) Databank, an electronic repository of health data hosted by Swansea University [26, 27]. SAIL aims to take advantage of routinely collected person-level electronic data for health and social research. SAIL was queried for persons having an ICD code of 340 or G35x (MS) during the period 2002 to 2013. Using ICD codes for case ascertainment has a sensitivity between 85–92.4 and a specificity between 55.9–92.6 [28]. The Patient Episode Database for Wales (PEDW) database in SAIL has 100 percent coverage of inpatient and outpatient (daycase) hospital visits in Wales. Since the UK clinical guidelines of 2003 [29]have required that MS diagnosis be made by a specialist neurologist on the basis of lesions, the PEDW is a trustworthy source of confirmed MS cases. PEDW does not capture MS cases diagnosed in primary care. PEDW has individual-level data, obscuring the values of certain variables (e.g. date of birth becomes week of birth) for confidentiality. Each person has a unique identifier (called an “anonymous linking field”) which matches one-to-one with a valid NHS number [30]. We extracted from PEDW the gender, week of birth, and Lower Layer Super Output Area of residence (LSOA) of patients with MS. In the UK, an LSOA is a geographic area with a mean population of 1,500. There are 1,909 LSOAs in Wales.

Mid-year population estimates from 2002 to 2013 for all LSOAs in Wales were downloaded from the Small Area Population Estimates of the ONS website. We summed these age-and -gender-specific mid-year estimates in order to estimate the person-years contributed by each LSOA. This served as our denominator for calculating incidence rate.

The UK Office of National Statistics (ONS) also has a list of LSOAs designated as coastal communities (n = 422, for Wales). Accordingly, we coded all LSOAs in this list as “1” and the rest as “0”. There were LSOAs that changed (either merged or split) between the UK Census 2001 and 2011 so we harmonized the codes using a cross-reference file provided by the ONS.

For sunshine levels, we used the 30-year average sunshine hours per day at each LSOA for the period 1961 to 1990. The Met Office does not provide sunshine levels for each LSOA. Instead, the Met Office divides UK land area into 440 grid boxes, each measuring 25 square kilometres [31]. The method for constructing these grids is described elsewhere [32]. These grid boxes are associated with rotated longitude/latitude values which we translated into real longitude/latitude using a reference table at this web page: http://ukclimateprojections-ui.metoffice.gov.uk/ui/docs/grids/prob_land_25km_rotated/index.php. Also, ONS has a file that indicates the population centroids (in longitude/latitude) for each LSOA. We then matched each population-weighted LSOA to the appropriate grid box based on the shortest haversine distance. Unlike Euclidean distance, haversine takes into account the earth’s curvature. In summary, the MET Office provided sunshine hours for grid boxes which we matched to LSOAs provided by the ONS using the longitude/latitude as the merge field.

### Statistical Analysis

We calculated age-standardized incidence by first creating five-year age groups and dividing the number of cases by the corresponding person-years in each LSOA. We collapsed the first two groups (0 to 10 years) due to small numbers of cases. Age-specific estimates were calculated separately for males and females. We finally standardized these rates to the 1976 European standard population. Age-standardization allows for the comparison of rates where the age structure of populations are not the same.

To examine whether MS is seasonal by birth, we compared the number of MS patients born in any given month with their counterparts in the general population of England and Wales. Because month of birth was not tallied separately by the UK Office of National Statistics (ONS) until the 1960s, we had to use combined figures for both nations. Furthermore, the ONS only started recording month of birth from 1938. As a result, our month of birth analysis was restricted to 2,927 MS patients (82 percent) who were born since 1938. We performed chi-square tests to test whether the pattern of MS births resembled the general population. To keep the family-wise error rate at .05, we required that each month’s chi-squared result be significant at an alpha of .004.

We examined the influence of the environment by aggregating MS cases per LSOA (if any) over the study period. This was our dependent variable. Our substantive predictors were hours of sunshine per day, latitude, longitude, and whether the LSOA was a coastal city. For ease of interpretation, we centred latitudes on Aberystwyth (latitude = 52.42) in the north/south dimension. We also centred longitudes on Carmarthenshire (longitude = -4.26) in the east/west dimension. Mid-year population estimates were used as an offset term in order to relate the number of MS cases per LSOA with its mid-year population for each year. We explored various interactions among predictors and used the pseudo-R squared statistic as a criterion for selecting the best model.

## Results

**Fig 1:**
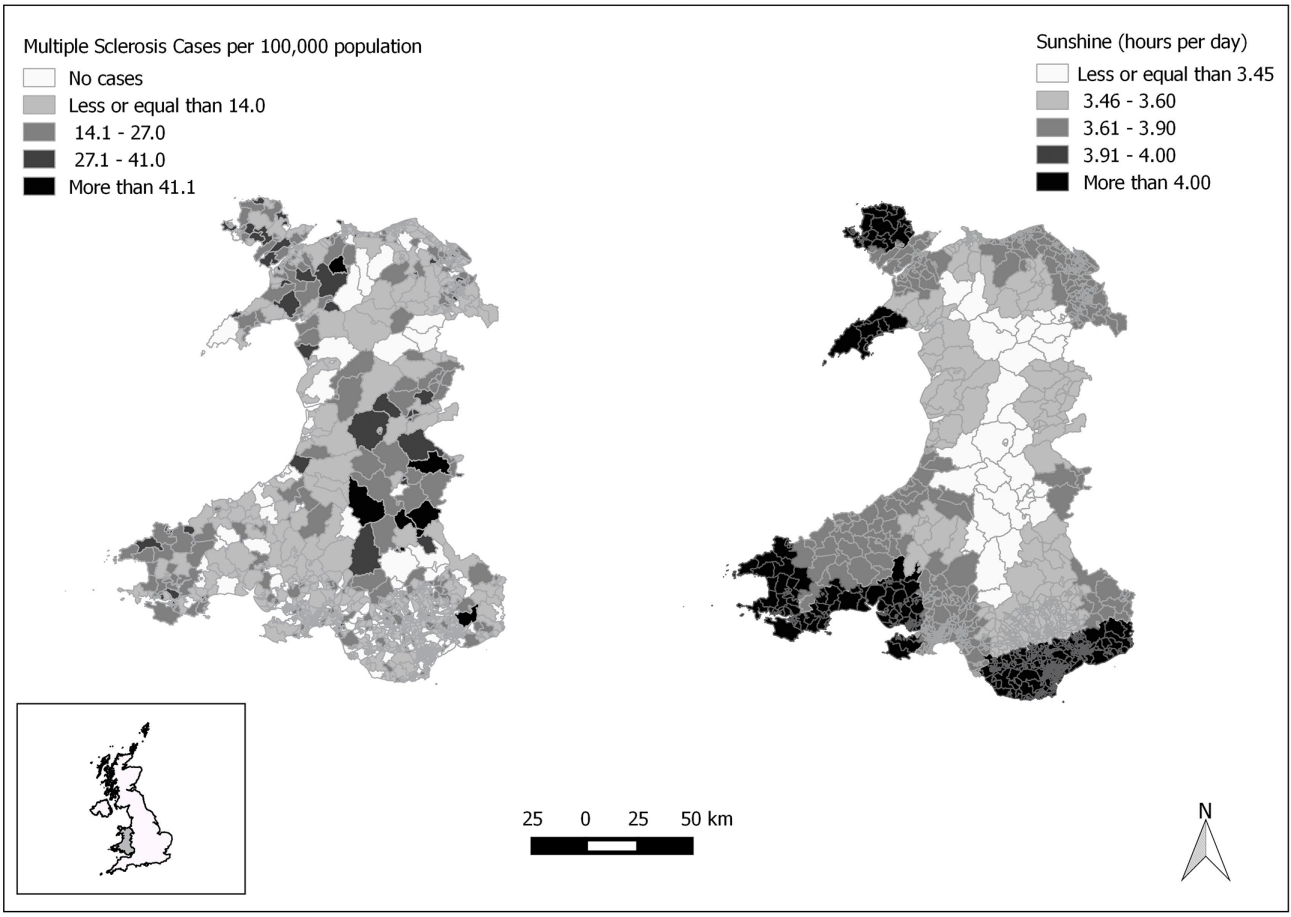
Average annual incidence of MS (2002 to 2013) and Average Sunshine Hours per day in Wales, UK. Left panel: Incidence by LSOA. Right panel: Sunshine hours per day from 1961–1990. Inset: Wales location within the UK.

From 2002 to 2013, there were 1,256 new MS cases among men and 2,301 new cases among women. The average annual incidence rate per 100,000 people was 8.14 (95% CI: 7.69–8.59) for males and 12.97 (12.44–13.50) for females, for a sex ratio of 1.86:1 (female: male). For the total population, the combined average incidence rate was 9.10 (8.80–9.40) (Table 1).These figures are standardized to the age-distribution of the 1976 European population. The mean age at first hospital admission for males was 52.95 (SD: 19.94) years and 50.47 (SD: 18.32) for women. As expected, MS incidence in ages 0 to 10 for both sexes is low. In males, there is an increase starting around age 30 and around age 25 for females. For both sexes, the highest incidence is at age-group 85 and above, but this could be due to the aggregation of many ages or late diagnosis. The disease onset is likely to have occurred much earlier—we address this matter in the limitations section. See Table 1 for the sex-specific and combined MS incidence estimates. The distribution of cases and sunshine levels by LSOA (Fig 1).

**Table 1:** Average Annual Incidence of Multiple Sclerosis in Wales, by Sex and Age Group, from 2002 to 2013.

	Males			Females			Combined		
Age Group	New Cases	Person-Years	Age-specific rate**	New Cases	Person-Years	Age-specific rate**	New Cases	Person-Years	Age-specific rate**
0 to 9	9	2,092,636	0.43	8	1,985,087	0.4	17	4,077,723	0.42
10–14	19	1,139,767	1.67	11	1,083,276	1.02	30	2,223,043	1.35
15–19	25	1,209,624	2.07	26	1,155,986	2.25	51	2,365,610	2.16
20–24	32	1,208,228	2.65	60	1,177,370	5.1	92	2,385,598	3.86
25–29	46	1,052,498	4.37	117	1,045,740	11.19	163	2,098,238	7.77
30–34	75	1,056,610	7.1	158	1,084,615	14.57	233	2,141,225	10.88
35–39	116	1,162,755	9.98	272	1,209,146	22.5	388	2,371,901	16.36
40–44	109	1,251,252	8.71	282	1,302,072	21.66	391	2,553,324	15.31
45–49	124	1,215,787	10.2	290	1,260,590	23.01	414	2,476,377	16.72
50–54	116	1,156,919	10.03	244	1,194,131	20.43	360	2,351,050	15.31
55–59	133	1,158,074	11.48	192	1,192,054	16.11	325	2,350,128	13.83
60–64	98	1,088,416	9	155	1,123,714	13.79	253	2,212,130	11.44
65–69	86	918,417	9.36	120	966,922	12.41	206	1,885,339	10.93
70–74	76	737,121	10.31	94	826,237	11.38	170	1,563,358	10.87
75–79	49	567,655	8.63	83	715,409	11.6	132	1,283,064	10.29
80–84	44	378,833	11.61	47	577,891	8.13	91	956,724	9.51
85+	99	250,076	39.59	142	569,334	24.94	241	819,410	29.41

** Per 100,000 population

**Fig 2:**
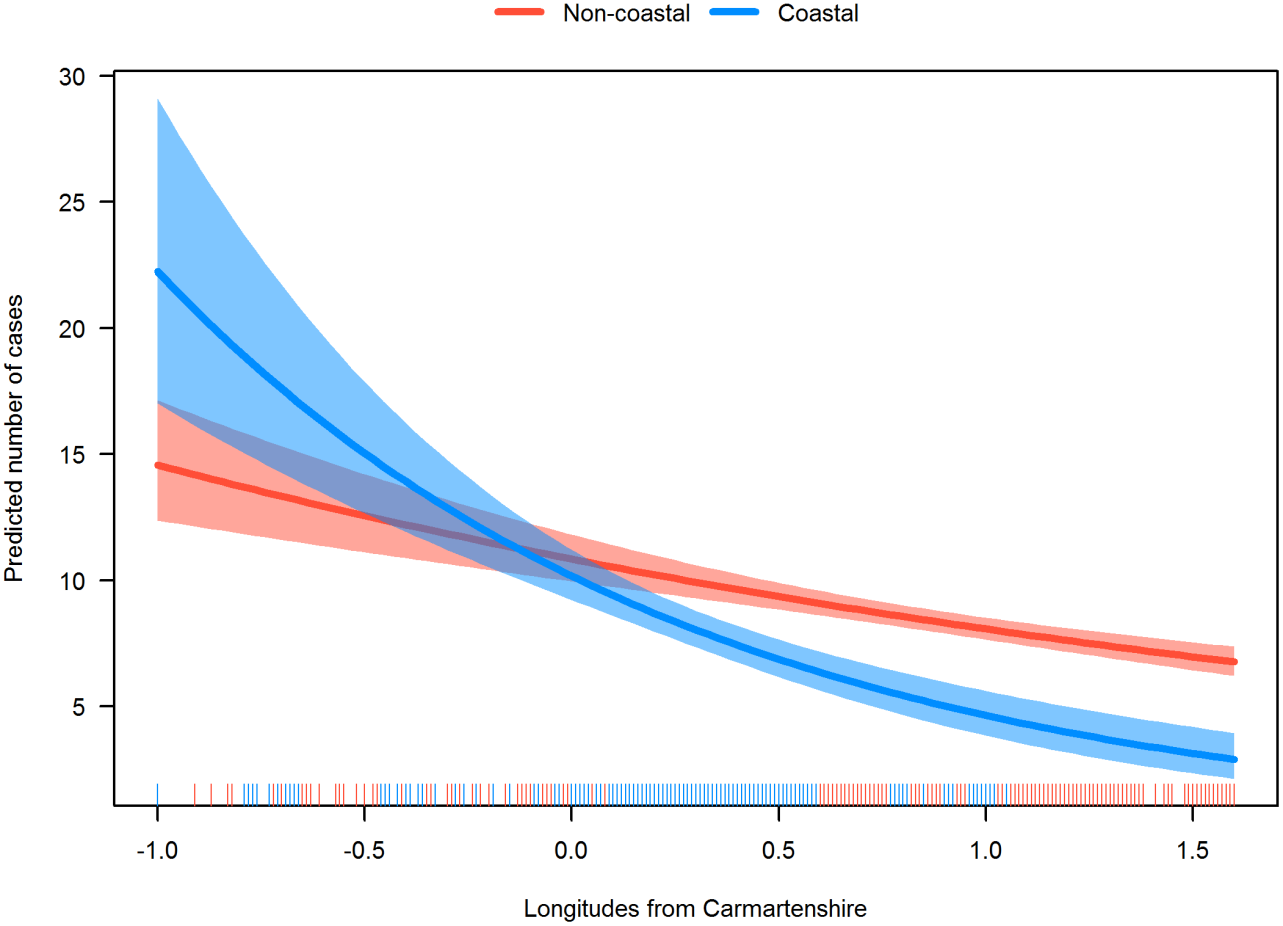
Coast × Longitude interaction in the incidence of multiple sclerosis by LSOA in Wales, UK. Rug plot on the x-axis indicates the distribution of coastal and non-coastal areas.

Month-by-month chi-square tests showed a 21 percent higher number of MS births in April (Observed to Expected Ratio: 1.21 (95% CI: 1.08–1.36)) as compared with the general population (χ^2^ = 10.99, df = 1, p < .001). No other month had birth numbers that differed significantly from expected (Table 2). The regression model for the count of MS cases by LSOA showed that MS incidence decreased by 25 percent for each additional hour of sunshine per day. For each additional latitude north of Aberystwyth, MS incidence increased by 37 percent. There was a significant *Coast × Longitude* interaction indicating that easterly coastal areas in Wales had lower MS incidence. For those living west of Carmarthenshire, living in a coastal area did not make a difference in incidence rates (Table 3 and Fig 2).

**Table 2:** Month of birth from 1938 to 2005, General Population vs People with MS in Wales.

Month	All England and Wales births	Births of People with MS in Wales		Observed-to-Expected Ratio (95% CI)	Chi-square	P
		Observed	Expected			
January	3,938,576	238	244	0.98 (0.86–1.11)	0.15	0.70
February	3,693,432	221	229	0.97 (0.84–1.10)	0.28	0.60
March	4,162,616	230	258	0.89 (0.78–1.01)	3.04	0.08
April	3,973,205	298	246	1.21 (1.08–1.36)	10.99	<.001
May	4,146,640	268	257	1.04 (0.92–1.18)	0.47	0.49
June	3,969,042	242	246	0.98 (0.86–1.12)	0.07	0.80
July	4,062,277	254	252	1.01 (0.89–1.14)	0.02	0.90
August	3,953,452	244	245	1.00 (0.88–1.13)	0.00	0.95
September	3,942,578	265	245	1.08 (0.96–1.22)	1.63	0.20
October	3,882,108	228	241	0.95 (0.83–1.08)	0.70	0.40
November	3,664,537	220	227	0.97 (0.85–1.11)	0.22	0.64
December	3,802,065	219	236	0.93 (0.81–1.06)	1.22	0.27

**Table 3:** Quasi-poisson regression model of MS births by LSOA Characteristic.

Explanatory Variable	Incidence Rate Ratio	95% CI
Average daily sunshine hours	0.75	0.63–0.91
Latitudes north of Aberystwyth	1.37	1.30–1.45
Longitudes east of Carmarthenshire	0.74	0.68–0.81
Coastal area (yes/no)	0.94	0.83–1.05
Longitudes east of Carmartenshire x Coastal Area	0.61	0.49–0.77

## Discussion

The main finding of this study is that Wales has an incidence rate of MS similar to that of Scotland. Higher daily sunshine hours was inversely associated with MS. Our results also supported the latitude hypothesis, with the number of cases increasing as one moves north within Wales itself. Residing in a coastal area was associated with lower MS incidence in more easterly areas. There was a higher than expected number of MS births for April, suggesting that maternal sunshine exposure during pregnancy is involved.

In comparison to other studies, our all-Wales age-standardized incidence rates are considerably higher than in previous UK studies [4, 33]where the estimates for females ranged from 7.2 to 11.52 and for males 3.1 to 4.84 per year per 10^5^ population [3, 34]. A previous study of average annual MS incidence in South East Wales during 1985 to 2005 reported 1.65 and 4.66 for males and females, respectively per 10^5^ population. Various areas in Scotland have reported combined sexes incidence rates of 5.7 in Glasgow, 7.2 in Tayside, 10.1 in the Border Region, and 12.2 in the Lothian Region per year per 10^5^ population [34–36]. It is striking that our sex-specific incidence rates for Wales are within the same range as the estimates for Scotland. Our incidence rates for Wales are slightly higher than for those in France where the figures are 7.5 for males and 10.4 for females [37]. Although the latter is consistent with the latitude gradient, the similarity of the incidence rates in Wales and Scotland is quite unexpected.

That the highest numbers of MS cases for both males and females occurred in the 85 and over age group is surprising. This could be an artefact of the higher hospital visits in this subgroup and/or collapsing more than five age groups in the category. One possibility is that although MS was pre-existing in some of these older people, a diagnosis was made only upon visiting the hospital—which tends to be more likely at older ages. The change in UK guidelines in 2003 may have contributed to the high MS incidence in the 85 and above age group. The other possibility is that there truly is a spike in MS incidence in these ages during 1990 to 2010 [3]. Unfortunately, we cannot determine which of the two possibilities is true.

The finding that MS cases are 20 percent higher in April has been previously reported in Scotland, the UK as a whole, Italy, and Finland [11, 38]. However, contrary to previous results, we neither found higher births in May or lower births in October or November[10, 39]. Dobson and colleagues proposed that month of birth reflects the availability of vitamin D in the prenatal environment [11]. Vitamin D helps tune the foetal immune system by suppressing inflammatory cytokines and promoting self-tolerance [7]. MS represents a disturbance of the immune system in which T-cells attack myelin protein [40].The winter months in the UK are from December to February, so those born in April would have been in their 5^th^ to 7^th^ months of gestation. It would be important to further examine if maternal vitamin D sufficiency is more critical in certain months of gestation.

Above 52 degrees latitude (roughly, Cambridge or Aberystwyth, UK), there is insufficient UV light for vitamin D synthesis during the winter months [29]. In this regard, proposals have been put forward for dietary supplementation [11]. The ecological nature of our study design makes us unable to interpret the link of more easterly coastal residence with lower MS births. A future study, with measures of individual fish consumption, would be able to comment on this finding. The evidence regarding dietary supplementation is inconsistent. Eating fish three times a week or more in childhood and adolescence was found protective for MS [30]. Munger and colleagues [41]pooled two large prospective cohorts of nurses (n > 150,000). They found that >400 IU of vitamin D from supplements was protective for MS. In Canada, the association of serum 25OHD levels and dietary vitamin D intake (from milk and multivitamins) was studied [42]. No significant association was found. More studies are required as to whether the risks of fortifying food with vitamin D are outweighed by its benefits. Thus far, small clinical studies have shown that vitamin D supplementation is safe and these results support large randomized trials [6].

As with all studies, the present one is subject to several limitations. First and foremost, the present study is ecological in design and did not include individual level variables other than week of birth and gender. While coastal residence could serve as population-level proxy for seafood consumption, the lack of person-level dietary information precludes an interpretation. Secondly, the present study relied on a hospital-based register. This means that persons with MS who do not visit the hospital go undetected. We have chosen to err on the side of underestimation by not using the general practice register for two reasons. UK clinical guidelines since 2003 require that patients suspected of having MS be referred to a specialist neurologist on the basis of CNS lesions [29]. Furthermore, a previous study using the general practice database noted that the diagnosis of multiple sclerosis in the General Practice Research Database has not been validated [3]. In analysing MS months of birth, we used the combined England and Wales births from 1938 to 2005. This could affect our estimate of April births if the birth pattern differed between England and Wales. The main strengths of our study are data from a hospital-based administrative register with 100 percent coverage of hospital visits in Wales and data on sunshine hours at the small area level.

## Conclusion

This is the first study of MS incidence covering all of Wales and we found an incidence rate comparable to that of Scotland. The variation in incidence is related to geographical factors that probably represent levels of vitamin D.

## Supporting Information

S1 File Minimal Data to Replicate the Analysis. This is a compressed Microsoft Excel file containing lower layer super output areas in Wales, population counts by age and gender groups, sunshine levels, and an indicator for coastal status.
(DOCX)
