# Supplementary material for: Sunshine, Sea, and Season of Birth: MS Incidence in Wales
Source: PLoS One. 2016 May 16;11(5):e0155181. doi: 10.1371/journal.pone.0155181 (PMC4868284; doi:10.1371/journal.pone.0155181)
Supplement: S1 File — This is a compressed Microsoft Excel file containing lower layer super output areas in Wales, population counts by age and gender groups, sunshine levels, and an indicator for coastal status. (DOCX) [file pone.0155181.s001.docx]

**Supporting Information**

**S1 File. Minimal Data to Replicate the Analysis.** This is a compressed Microsoft Excel file containing lower layer super output areas in Wales, population counts by age and gender groups, sunshine levels, and an indicator for coastal status.
